# 479. Real-world Evaluation of SARS-CoV-2 Monoclonal Antibodies in Solid Organ Transplant Recipients

**DOI:** 10.1093/ofid/ofab466.678

**Published:** 2021-12-04

**Authors:** Kristen Zeitler, Nicholas Piccicacco, Lyndsey Bowman, Jose Montero, Margarita Cancio, Sally Houston, Kami Kim

**Affiliations:** 1 Tampa General Hospital, Tampa, FL; 2 University of South Florida Morsani College of Medicine, Tampa, FL; 3 Infectious Disease Associates of Tampa Bay, Tampa, Florida; 4 University of South Florida, Tampa, Florida

## Abstract

**Background:**

Patients with COVID-19 infection at highest risk for poor outcomes include immunocompromised patients, such as solid organ transplant (SOT) recipients. Monoclonal antibody (mAb) infusions were developed to promote passive immunity. Analysis of the first 200 patients who received SARS-CoV-2 mAb at our hospital showed a 27 % absolute reduction in hospitalization and emergency department (ED) visits. Understanding the role of SARS-CoV-2 mAb therapy in management of the SOT population is likely to inform decision making for these patients.

**Methods:**

We conducted a retrospective chart review of SOT patients diagnosed with COVID-19 who received mAb therapy between 11/18/20 and 04/26/21. Patients were excluded if they were < 18 years of age or if they weighed < 40 kg. We compared those patients who were hospitalized or visited the ED within 29 days of mAb therapy to those who recovered without further visits to our hospital.

**Results:**

A total of 50 SOT patients receiving mAb therapy were included in this analysis. Bamlanivimab was given to 33 patients, while 9 patients received bamlanivimab/etesevimab and 8 patients received casirivimab/imdevimab. Twelve (24 %) patients were hospitalized or visited the ED within 29 days of mAb therapy; 38 patients did not. These 2 groups did not significantly differ by age, gender, body mass index, time from SOT, or other risk factors for severe COVID-19 illness per FDA Emergency Use Authorization guidance. Both groups were primarily made up of kidney transplant recipients (66.7 % and 68.4 %, respectively). Significantly more patients in the hospitalization/ED group were receiving antimetabolites as part of their immunosuppression (IS) regimen prior to COVID-19 diagnosis (100 % vs 68.4 %, p = 0.047). Patients in the hospitalization/ED group received mAbs within a median of 6 days (IQR 3.8) of symptom onset compared to 4 days (IQR 4) (p = 0.006).

**Conclusion:**

SOT recipients were more likely to be hospitalized or visit the ED due to COVID-19 after mAb if they were receiving antimetabolite IS or received mAb later after symptom onset. These data stress the importance of early mAb administration in all SOT patients, particularly in those on antimetabolite therapy.

**Disclosures:**

**Lyndsey Bowman, PharmD**, **Veloxis Pharmaceuticals** (Advisor or Review Panel member, Speaker’s Bureau) **Kami Kim, MD**, **Regeneron Pharmaceuticals Inc.** (Scientific Research Study Investigator)**Sanford Guide** (Other Financial or Material Support, Editorial Board Member)

